# Artificial Intelligence Literacy and Subjective Workload Among Clinical Nurses: A Cross-Sectional Study

**DOI:** 10.3390/healthcare14193155

**Published:** 2026-09-23

**Authors:** Che-Wei Yu, Cheng-Chia Yang

**Affiliations:** 1 Department of Nursing, Asia University Hospital, Taichung 413505, Taiwan; 050932@tool.caaumed.org.tw; 2 Department of Healthcare Administration, College of Medical and Health Science, Asia University, Taichung 413305, Taiwan

**Keywords:** artificial intelligence literacy, nursing workload, subjective workload, NASA-TLX

## Abstract

**Highlights:**

**What are the main findings?**

- Higher AI literacy is associated with lower temporal demand and frustration among clinical nurses.
- The associations are not uniform across workload dimensions, with no robust relationships observed for mental demand, effort, or performance.

**What are the implications of the main findings?**

- AI literacy is associated with selected dimensions of nurses’ subjective workload rather than workload uniformly.
- These associations should be interpreted alongside workflow, training, and organizational factors and examined further in longitudinal and intervention studies.

**Abstract:**

**Background**: Although artificial intelligence (AI) is increasingly being integrated into nursing practice, little is known about the association between nurses’ AI literacy and their subjective workload. This study examines the associations between three dimensions of AI literacy—technical understanding, critical appraisal, and practical application—and overall and dimension-specific subjective workload among clinical nurses. **Methods**: A cross-sectional survey was conducted among 305 clinical nurses recruited through convenience sampling from two hospitals in central Taiwan. AI literacy was assessed using the self-report Scale for the Assessment of Non-Experts’ AI Literacy (SNAIL), with responses rated on a five-point Likert scale. Subjective workload was assessed using the self-report NASA Task Load Index (NASA-TLX). Pearson correlation and hierarchical multiple linear regression analyses were performed, adjusting for relevant demographic and work-related characteristics. **Results**: Higher technical understanding, critical appraisal, and practical application were each associated with lower temporal demand and frustration, and these associations remained statistically significant after correction for multiple testing. More limited associations were observed for physical demand and the overall NASA-TLX workload score, while no robust associations were found for mental demand, effort, or performance. **Conclusions**: The assessed dimensions of AI literacy are found to be associated with specific dimensions of nurses’ subjective workload, rather than with workload uniformly, with the clearest relationships observed for temporal demand and frustration. Further longitudinal and intervention studies are needed to determine whether improvements in AI literacy are accompanied by meaningful changes in nurses’ workload experiences.

## 1. Introduction

Artificial intelligence (AI) applications are increasingly being integrated into nursing practice, including for clinical decision support, patient monitoring, documentation, and administrative tasks [1,2,3]. However, concerns remain regarding the reliability and consistency of AI-generated outputs, including inaccurate or fabricated information [1,2,3]. The implementation of AI also raises issues related to bias, data privacy, transparency, and accountability, while the responsibility for clinical decisions continues to rest with healthcare professionals [1,2,4]. Safe use of AI therefore requires nurses to understand its capabilities and limitations, and to evaluate its outputs in conjunction with clinical judgment [1,2,4].

AI literacy broadly refers to the knowledge and judgment required to understand, evaluate, and appropriately use AI [5,6]. Existing frameworks conceptualize AI literacy as a multidimensional construct encompassing technical knowledge, recognition of system capabilities and limitations, critical evaluation of outputs, practical application, and ethical judgment [5,6]. Existing AI literacy instruments also vary in their conceptual coverage and measurement approaches [7], underscoring the importance of considering distinct dimensions of AI literacy rather than treating it as a single undifferentiated construct.

Recent nursing studies have begun to examine nurses’ AI literacy and AI readiness. Perioperative nurses in Türkiye reported moderate self-rated AI literacy [8], while surgical nurses reported above-average medical AI readiness, with AI literacy being positively associated with readiness [9]. A survey conducted in Germany found limited AI-related knowledge and minimal workplace exposure to AI among many nurses [10]. A recent scoping review identified foundational knowledge, technical cognition, application skills, perceived utility, readiness, ethical awareness, critical thinking, and innovation as recurring elements of AI literacy in the nursing context [11]. However, existing nursing research has largely focused on the level of AI literacy, readiness, attitudes, and adoption intentions [8,9,10,11,12,13] and, although recent studies have begun to extend this literature toward occupational and psychological outcomes—including psychological capital, occupational resilience, and engagement in AI-supported nursing work [14,15,16]—the relationships between different dimensions of AI literacy and nurses’ subjective workload remain largely unexplored.

The nursing workload is a multidimensional construct shaped by patient complexity, staffing adequacy, care demands, and organizational conditions [17,18,19,20]. The NASA Task Load Index (NASA-TLX) assesses six subjective workload dimensions; namely, mental demand, physical demand, temporal demand, perceived performance, effort, and frustration [21]. Studies using the NASA-TLX among nurses have shown that these dimensions may reflect distinct aspects of clinical work [22,23], suggesting that examining them separately may yield more information than treating the nursing workload as a single, undifferentiated construct.

Research in the adjacent fields of digital health and nursing informatics offers indirect evidence that user competence may be related to the perceived technology-related work demand. In a nationwide Finnish survey of 3607 registered nurses, lower e-Care competence was associated with greater time pressure and psychological distress [24]. Another Finnish study found that higher nursing informatics competence was associated with lower stress and psychological distress among newly graduated nurses, although this association was not observed among experienced nurses [25]. More recent studies have shown that insufficient training during electronic health record implementation was associated with greater information-system-related stress and time pressure [26]. Higher digital literacy has also been associated with lower work stress among emergency nurses [27], while technology competence was found to moderate indirect pathways linking technostress creators with burnout and job satisfaction [28]. Collectively, these findings suggest that nurses’ digital and informatics competencies may be related to their experience of time pressure and technology-related strain.

It should be noted, however, that digital and informatics competencies are not equivalent to AI literacy. AI literacy additionally involves AI-specific knowledge, understanding of the capabilities and limitations of AI systems, critical evaluation of AI-generated outputs, and how to appropriately apply AI [5,6,11]. A more specific conceptual basis for considering AI literacy in relation to subjective workload can be derived from the Job Demands–Resources (JD–R) framework. In particular, this framework distinguishes job demands—which require sustained physical or psychological effort—from resources—which help employees to meet their work demands and maintain effective functioning [29]. Extensions of this model further recognize personal resources, such as self-efficacy and optimism, as individual capacities that support employees in coping with demanding work conditions [30]. From this perspective, AI literacy may be conceptualized as an AI-specific personal resource that is relevant to nurses’ adaptation to increasingly AI-integrated work environments, rather than as another form of general digital competence.

Recent nursing evidence supports this resource-based conceptualization. Qin et al. [15] found that higher AI literacy among clinical nurses was associated with greater psychological capital and occupational resilience. Similarly, Lin et al. [16] applied the JD–R framework to nurses using an AI-supported documentation system and conceptualized AI literacy as a personal resource. They found that AI literacy was positively associated with engagement in nursing administrative tasks, but was not significantly associated with general job stress [16]. Together, these findings suggest that AI literacy may be relevant to nurses’ adaptation to work environments in which AI technologies are increasingly being introduced, although such an association may differ across work-related outcomes.

Against this background, the resource-based perspective may help to explain why AI literacy could be differentially associated with specific dimensions of subjective workload. Temporal demand in the NASA-TLX reflects perceived time pressure during task performance [21]. Previous nursing studies have shown that lower e-Care or informatics competencies are associated with greater perceived time pressure and technology-related strain [24,25,26]. Although these competencies are distinct from AI literacy, these findings indicate that competencies relevant to the technological work environment may be associated with how strongly time-related demands are experienced. As AI technologies are increasingly being introduced into healthcare settings, AI literacy may represent an AI-specific personal resource relevant to nurses’ responses to emerging technology-related work demands.

A related resource-based rationale may apply to frustration. Within the NASA-TLX, frustration reflects the affective strain experienced during task performance, including feelings such as stress, irritation, discouragement, and annoyance [21]. In the context of the JD–R framework, it has been proposed that demanding work conditions can generate psychological strain when employees must expend sustained resources to meet those demands [29]. In nursing research, psychological capital has been inversely associated with burnout and emotional exhaustion [31], while Qin et al. [15] found that higher AI literacy was associated with greater psychological capital and occupational resilience. These findings provide a conceptual basis for examining whether AI literacy is also associated with the affective dimension of subjective workload represented by frustration. However, burnout and emotional exhaustion are distinct from the task-related frustration, as assessed via the NASA-TLX; therefore, this evidence should be regarded as supporting rather than direct.

Taken together, previous research on digital and informatics competence provides indirect evidence that technology-related competence may be associated with perceived time pressure and psychological strain, while emerging AI-specific nursing research suggests that AI literacy may function as a distinct personal resource which is relevant to occupational adaptation in increasingly AI-integrated healthcare environments. Whether there exist associations between AI literacy and nurses’ multidimensional subjective workload—as well as whether such associations differ across workload dimensions—remains unclear.

To our knowledge, the relationships between various dimensions of AI literacy and the six NASA-TLX dimensions of subjective workload have not been previously examined among nurses. The present study was therefore designed to be exploratory rather than confirmatory, and no formal directional hypotheses were specified. Accordingly, this cross-sectional study examined the relationships between nurses’ self-reported AI literacy and subjective workload, rather than the workload experienced during the use of a specific AI system or intervention. AI literacy was assessed using the Scale for the Assessment of Non-Experts’ AI Literacy (SNAIL), which comprises technical understanding, critical appraisal, and practical application dimensions [32], while subjective workload was assessed across the six NASA-TLX dimensions [21]. This study aimed to (1) describe AI literacy and subjective workload among clinical nurses in Taiwan; (2) assess the associations between the dimensions of AI literacy and both overall subjective workload and the six NASA-TLX dimensions; and (3) determine whether further consideration of the dimensions of AI literacy explains additional variance in the overall Raw TLX score and individual workload dimensions after adjustment for relevant background characteristics.

## 2. Materials and Methods

### 2.1. Study Design and Setting

The study was conducted at two regional hospitals within the same healthcare system in central Taiwan. Hospital A had 396 beds and approximately 480 nursing personnel, whereas Hospital B had 460 beds and approximately 646 nursing personnel. Data were collected from 1 to 31 March 2026. The inclusion criteria were as follows: (1) aged 18 years or older; (2) currently performing clinical nursing duties at one of the participating hospitals; (3) at least one year of clinical nursing experience; and (4) willingness to participate in the study. A non-probability convenience sampling approach with voluntary participation was used. At each participating hospital, eligible clinical nurses were approached with the assistance of the nursing department. The researchers provided information regarding the study’s purpose, eligibility criteria, study procedures, voluntary participation, and confidentiality. Approximately 467 eligible nurses were approached across the two participating hospitals. Nurses who met the eligibility criteria and were interested in participating accessed the anonymous web-based questionnaire independently via a QR code provided by the researchers. The first page of the questionnaire presented a study information sheet explaining the voluntary nature of participation and the measures used to protect confidentiality. Participants could discontinue the questionnaire at any time before submission without penalty, and no personally identifiable information was collected. Under the IRB-approved consent procedure, voluntary completion and submission of the questionnaire were considered to constitute informed consent. A total of 305 completed questionnaires were included in the analysis. As recruitment and questionnaire completion were anonymous and were not linked at the individual level, a precise response rate could not be determined. Based on the approximate number of nurses approached, the proportion of participating respondents was approximately 65%.

### 2.2. Measures

The questionnaire comprised three sections: (1) demographic, work-related, and technology-related characteristics; (2) AI literacy, assessed using the Scale for the Assessment of Non-Experts’ AI Literacy (SNAIL); and (3) subjective workload, assessed using the National Aeronautics and Space Administration Task Load Index (NASA-TLX).

The first section collected demographic, work-related, and technology-related characteristics. Demographic characteristics included gender, age, educational level, and marital status. Work-related characteristics included nursing clinical ladder level, shift type, supervisory status, involvement in administrative duties, geographic region, primary clinical unit, and years of clinical nursing experience. Technology-related characteristics included self-rated acceptance of technology, willingness to use new technologies at work, familiarity with AI, and prior experience using AI-assisted tools.

The second section assessed self-reported AI literacy using an adapted Traditional Chinese version of the SNAIL [32]. The scale consists of 31 items across 3 dimensions: technical understanding (14 items), critical appraisal (10 items), and practical application (7 items). The original English version was translated into Traditional Chinese and back-translated into English by a professional translation service, and discrepancies were reviewed by the research team to ensure semantic equivalence. The original seven-point response format was adapted to a five-point Likert scale ranging from 1 (strongly disagree) to 5 (strongly agree).

The choice of a five-point response format was informed by previous methodological evidence. An experimental comparison revealed that five- and seven-point scales produced the same mean score after rescaling, with little difference in other distributional characteristics, including variation, skewness, and kurtosis [33]. A Monte Carlo simulation study further confirmed that reliability and validity increased with the number of response categories, with the optimal range being between four and seven categories and little additional psychometric gain beyond seven [34]. In addition, a five-point Likert response format has been used in previous research for selected items adapted from the SNAIL [35].

A mean score was calculated for each dimension, with higher scores indicating higher levels of self-reported AI literacy, and the scores for the three dimensions were analyzed as continuous variables. In the present sample, Cronbach’s alpha was 0.958 for technical understanding, 0.937 for critical appraisal, and 0.890 for practical application.

The third section assessed subjective workload during the preceding month using the NASA-TLX [21], which comprises six dimensions: mental demand, physical demand, temporal demand, performance, effort, and frustration. Participants were instructed to consider their routine clinical nursing work during the preceding month and rate each dimension from 0 to 100. The one-month recall period was used to capture participants’ overall perception of routine clinical workload across recent work experiences, rather than workload associated with a single shift or isolated task. Higher scores indicated greater mental demand, physical demand, temporal demand, effort, and frustration; for the performance dimension, higher scores indicated poorer perceived performance. While the original NASA-TLX includes a pairwise weighting procedure to derive an overall weighted workload score [21], the Raw TLX approach was used in the present study (i.e., no pairwise weights were applied). Consistent with previous applications, the unweighted Raw TLX score was calculated as the arithmetic mean of the six dimension ratings [21,22]. The six dimensions were also analyzed separately, as combining all six ratings into a single score may not fully capture the multidimensional nature of nursing workload [23]. As each NASA-TLX dimension was assessed using a single rating, dimension-specific internal consistency coefficients are not applicable here.

### 2.3. Sample Size Determination

An a priori sample size calculation was performed using G*Power 3.1 for hierarchical multiple regression (fixed model, R^2^ increase). Given the limited prior evidence directly quantifying the association between AI literacy and nurses’ subjective workload, a relatively small incremental effect size (f^2^ = 0.05) was assumed for the a priori calculation, with an alpha level of 0.05 and statistical power of 0.80. For sample size planning, the three AI literacy dimensions were conservatively treated as the tested predictor set together with the prespecified demographic and work-related covariates. After dummy coding of categorical covariates, this corresponded to 3 tested predictors and 16 total predictor parameters. Under these assumptions, the minimum required sample size was 223 participants. Allowing for an anticipated 20% rate of incomplete or unusable responses, the target sample size was approximately 279 participants. The final analytic sample comprised 305 nurses, exceeding the planned minimum sample size.

### 2.4. Covariates

Six covariates were included consistently across all adjusted models; namely, gender, years of clinical nursing experience, primary clinical unit, shift type, supervisory status, and involvement in administrative duties. These variables were prespecified based on published evidence and their clinical relevance to nurses’ subjective workload, and no data-driven variable-selection procedure was used. Gender and clinical experience have previously been associated with the NASA-TLX workload among nurses [22,36], whereas clinical setting and shift-related characteristics may reflect differences in patient complexity, staffing conditions, work schedules, and workload demands [20,36,37]. Supervisory and administrative responsibilities were included because they may capture the impact of additional coordination, oversight, documentation, personnel management, and operational duties [38,39].

### 2.5. Statistical Analysis

Data were analyzed using IBM SPSS Statistics version 24. Categorical variables are summarized as frequencies and percentages, whereas continuous variables are summarized as means and standard deviations. Pearson correlation coefficients were calculated to examine the bivariate associations between the three AI literacy dimensions and the overall Raw TLX score and the six NASA-TLX dimension scores. For each of the seven workload outcomes, three separate hierarchical multiple linear regression models were fitted—one for each AI literacy dimension. In Model 1, gender, years of clinical nursing experience, primary clinical unit, shift type, supervisory status, and involvement in administrative duties were entered as covariates. In Model 2, one AI literacy dimension (technical understanding, critical appraisal, or practical application) was added separately while retaining the same covariates; thus, the regression coefficients for each AI literacy dimension were adjusted for the background covariates but not mutually adjusted for the other AI literacy dimensions. The three AI literacy dimensions were examined in separate adjusted models because the primary objective was to characterize the association of each dimension with workload separately, rather than to estimate the unique contribution of each dimension after mutual adjustment. Changes in explained variance from Model 1 to Model 2 were assessed using ΔR^2^. These six covariates were selected based on previous studies and their conceptual relevance to nurses’ workloads. In additional sensitivity analyses, the models were further adjusted for age, educational level, and previous AI tool experience to assess the robustness of the primary findings. To account for multiple testing, the Benjamini–Hochberg procedure was applied across the 21 adjusted AI literacy–workload associations to control the false discovery rate at 5%. Potential common method variance was assessed using Harman’s single-factor test, in which the 31 SNAIL items and 6 NASA-TLX items were entered into an unrotated principal component analysis [40]. All tests were two-sided, with statistical significance set at *p* < 0.05.

### 2.6. Ethical Considerations

The study protocol was reviewed and approved by the Central Regional Research Ethics Committee, China Medical University, Taichung, Taiwan (approval no. CRREC-115-007). All participants were provided with information regarding the study purpose, procedures, voluntary participation, confidentiality, and their right to discontinue the questionnaire before submission. Under the IRB-approved consent procedure, voluntary completion and submission of the anonymous questionnaire were considered to indicate informed consent. No personally identifiable information was collected, and all data were handled and stored in accordance with the approved research protocol.

## 3. Results

### 3.1. Participant Characteristics

A total of 305 clinical nurses were included in the analysis. The sample was predominantly female (87.9%), and the largest age group was 30–39 years (38.0%), followed by 18–29 years (30.5%). Just over one-third of participants (37.7%) held a university degree, and 50.2% were married or cohabiting. In terms of professional characteristics, 54.4% worked fixed day shifts, 84.3% held non-supervisory positions, and 65.6% reported involvement in administrative duties. Inpatient care units (38.4%) and special clinical units (33.8%) accounted for the largest proportions of primary work settings, and 34.4% of participants had 6–10 years of clinical nursing experience. Just over half of the participants (52.1%) reported prior experience using AI-assisted tools (Table 1).

### 3.2. AI Literacy and Subjective Workload

Among the three AI literacy dimensions, technical understanding had the lowest mean score (M = 2.98, SD = 0.83), falling slightly below the midpoint of the five-point scale. Critical appraisal (M = 3.37, SD = 0.78) and practical application (M = 3.34, SD = 0.77) had somewhat higher mean scores. Internal consistency was high across all three dimensions, with Cronbach’s alpha values of 0.958, 0.937, and 0.890, respectively. The mean overall Raw TLX score was 74.45 (SD = 12.06). Among the six workload dimensions, effort had the highest mean score (M = 84.39, SD = 15.39), followed by mental demand (M = 81.20, SD = 18.55), physical demand (M = 80.77, SD = 19.07), and temporal demand (M = 79.43, SD = 20.58). The mean performance score was 63.88 (SD = 29.89), whereas frustration had the lowest mean score (M = 57.05, SD = 23.77). In Harman’s single-factor test, the first unrotated component accounted for 46.49% of the total variance, indicating that no single component accounted for the majority of the variance.

### 3.3. Correlations Between AI Literacy Dimensions and Subjective Workload

The three SNAIL dimensions were strongly intercorrelated (r = 0.810–0.881, all *p* < 0.001), indicating substantial shared variance among the dimensions (Table 2). Technical understanding showed a weak negative correlation with the overall Raw TLX score (r = −0.128, *p* = 0.025), while neither critical appraisal (r = −0.075, *p* = 0.190) nor practical application (r = −0.090, *p* = 0.119) reached statistical significance at the overall level.

At the NASA-TLX dimension level, however, all three AI literacy dimensions were consistently and negatively correlated with physical demand, temporal demand, and frustration. Technical understanding was negatively correlated with physical demand (r = −0.147, *p* = 0.010), temporal demand (r = −0.193, *p* < 0.001), and frustration (r = −0.146, *p* = 0.011); critical appraisal showed a similar pattern with physical demand (r = −0.133, *p* = 0.020), temporal demand (r = −0.148, *p* = 0.010), and frustration (r = −0.155, *p* = 0.007); and practical application likewise correlated with physical demand (r = −0.161, *p* = 0.005), temporal demand (r = −0.156, *p* = 0.006), and frustration (r = −0.156, *p* = 0.006).

Critical appraisal (r = 0.113, *p* = 0.048) and practical application (r = 0.129, *p* = 0.024) were weakly positively correlated with the performance score. No significant correlations were observed between the AI literacy dimensions and mental demand or effort (Table 2).

### 3.4. Adjusted Associations Between AI Literacy and Subjective Workload Dimensions

After adjustment for prespecified background covariates, the most consistent associations were observed for temporal demand and frustration (Table 3). Higher technical understanding, critical appraisal, and practical application were each associated with lower temporal demand (ΔR^2^ = 0.021–0.034; all *p* ≤ 0.012) and lower frustration (ΔR^2^ = 0.025–0.031; all *p* ≤ 0.005). In the primary analyses, all three AI literacy dimensions were also associated with lower physical demand (all *p* ≤ 0.025), while technical understanding was additionally associated with a lower overall Raw TLX score (B = −2.22, 95% CI [−3.95, −0.50], *p* = 0.012). No significant adjusted associations were observed for mental demand or effort. Practical application showed a small positive association with the performance score (B = 4.88, 95% CI [0.10, 9.66], *p* = 0.045), but this association did not remain statistically significant after BH-FDR correction (adjusted *p* = 0.087).

After applying the Benjamini–Hochberg false discovery rate correction across the 21 adjusted associations, 9 of the 11 associations with unadjusted *p* < 0.05 remained statistically significant. These comprised all associations of the three AI literacy dimensions with temporal demand and frustration, the associations of critical appraisal and practical application with physical demand, and the association of technical understanding with the overall Raw TLX score.

In additional sensitivity analyses further adjusting for age, educational level, and previous AI tool experience, the overall pattern of findings was broadly similar to that observed in the primary analyses. Associations with temporal demand and frustration remained statistically significant after additional adjustment (Table S1).

## 4. Discussion

AI literacy was most consistently associated with lower temporal demand and frustration among the clinical nurses in this study, and these associations remained significant after correction for multiple testing. Associations with physical demand and overall Raw TLX workload were more limited, and no robust associations were observed for mental demand, effort, or performance. Given the exploratory design of the study and the absence of prespecified directional hypotheses, these dimension-specific findings should be interpreted as exploratory rather than confirmatory.

Among the three AI literacy dimensions, technical understanding had the lowest mean score. This relative pattern is broadly consistent with Laupichler et al. [41], who similarly reported lower technical understanding than critical appraisal and practical application among medical students. However, because that study used the original seven-point response format, comparisons should be limited to the relative pattern across dimensions rather than absolute scores.

The three SNAIL dimensions were strongly intercorrelated (r = 0.810–0.881), indicating substantial shared variance among the dimensions. This pattern is consistent with previous validation evidence for the SNAIL, demonstrating substantial correlations among the three dimensions [32]. Therefore, the findings should be interpreted as associations with each AI literacy dimension considered separately, rather than as the unique contribution of each dimension after mutual adjustment.

The association with temporal demand deserves particular attention, as time pressure is an important component of nursing workload. Previous research in nursing informatics offers some support for this pattern; for example, lower e-Care competence has been associated with greater time pressure, while inadequate training regarding electronic health record systems has been linked to greater technology-related strain [24,26]. Although these studies examined broader digital competencies rather than AI literacy specifically, they provide a relevant point of comparison for the present findings. It should be noted that, in this study, workload was assessed in relation to routine clinical work rather than during the use of a specific AI system. Therefore, the observed association should not be interpreted as evidence that AI literacy or actual AI use reduces temporal demand during AI-assisted clinical work, and longitudinal and task-specific studies will be necessary to determine the associations of AI literacy and/or actual AI use with workload during specific AI-assisted tasks.

All three AI literacy dimensions were significantly associated with lower frustration, whereas no significant associations were observed for mental demand or effort. This distinction is noteworthy because frustration in the NASA-TLX reflects negative emotional responses experienced during task performance and is conceptually distinct from mental demand and effort [21]. Recent nursing research has also suggested that digital competence may be relevant to work-related strain; for example, higher digital literacy was associated with lower work stress among emergency nurses [27], while technology competence has been shown to shape nurses’ responses to technostress [28]. One possible explanation is that nurses with greater AI literacy may be better prepared to interpret and manage technology-related information, thereby experiencing less uncertainty or difficulty in deciding how to proceed; this may contribute to lower frustration without necessarily reducing mental demand or effort. However, as frustration was assessed in relation to overall clinical work rather than AI-specific tasks in the present study, this explanation remains hypothetical. The observed association should not be interpreted as evidence that AI competency or actual AI use directly reduces clinical frustration. The findings for physical demand were less consistent and warrant greater caution. Although all three AI literacy dimensions were associated with lower physical demand in the primary analyses, only critical appraisal and practical application remained statistically significant after correction for multiple testing. Unlike temporal demand and frustration, a direct pathway linking AI literacy to the physical requirements of nursing work is not readily apparent. Previous studies have shown that nurses’ physical workload is associated with patient complexity, nursing activity demands, staffing, and workflow-related characteristics [20,36]. The associations observed here may therefore partly reflect differences in work context or task composition that were not fully accounted for in the adjusted models. Even within similar clinical units, nurses may differ in patient acuity, nurse-to-patient ratios, staffing adequacy, direct-care intensity, task allocation, and unit-specific responsibilities, which can influence both perceived physical demand and opportunities to develop or apply AI-related competencies. Furthermore, reverse directionality cannot be ruled out; for example, nurses experiencing greater physical demand may have less time or energy to engage with emerging technologies. Further research is needed to determine whether these associations reflect differences in work context, reverse directionality, or other unmeasured factors. Accordingly, these findings should not be interpreted as evidence that improving AI literacy would directly reduce nurses’ physical workload.

Although several associations reached statistical significance, the observed bivariate correlations were weak (with absolute r values of approximately 0.13–0.19), and the incremental variance explained by the AI literacy dimensions was small (ΔR^2^ up to 0.034). This suggests that AI literacy may represent only one of multiple factors associated with nurses’ subjective workload, and the practical or clinical significance of these associations should therefore be interpreted cautiously. Whether these associations are large enough to be meaningful from a workforce or clinical management perspective remains to be established.

These findings carry some implications for nursing workforce development under the increasingly technology-mediated nature of clinical environments. Recent research has suggested that digital literacy and nursing informatics competencies may function as personal resources alongside organizational resources in shaping nurses’ work-related outcomes [42]. Consistent with this perspective, AI literacy should not be viewed as a stand-alone solution to nursing workload; rather, helping nurses to understand what AI can and cannot do, evaluate AI-related information critically, and apply it appropriately may be one component of broader organizational efforts to adapt to emerging technologies. Accordingly, nursing education and in-service training could include AI literacy content addressing foundational technical understanding, critical appraisal of AI-generated information, and context-appropriate applications; furthermore, such training should be integrated with workflow-specific guidance and organizational support, rather than viewed as a stand-alone strategy for reducing workload. Future longitudinal and intervention studies should examine whether improvements in AI literacy, together with appropriate training, workflow design, and organizational support, are accompanied by changes in temporal demand, frustration, and other dimensions of subjective workload. In such studies, structured AI literacy training on AI capabilities, limitations, and critical evaluation could be provided, allowing AI literacy, actual AI use, and subjective workload to be assessed before training, after training, and at follow-up.

Several limitations of the present study should also be acknowledged. First, the cross-sectional design precludes causal inference and establishment of the direction of the observed associations. In particular, reverse causality is possible; for example, nurses experiencing greater workload may have less time or opportunity to develop AI-related knowledge and skills. Second, both AI literacy and subjective workload were assessed concurrently using self-report measures, which may introduce common-method and response biases. Although Harman’s single-factor test showed that the first component accounted for 46.49% of the total variance—below the commonly used 50% criterion [40]—common-method bias cannot be completely excluded. In addition, because participants were recruited using convenience sampling and participation was voluntary, self-selection and non-response biases cannot be ruled out, as those who chose to participate may have differed from those who did not. Third, although professional translation and back-translation procedures were used and the adapted SNAIL demonstrated high internal consistency in the present sample, the Traditional Chinese version with the modified five-point response format was not subjected to separate formal cross-cultural or construct validation. Therefore, psychometric equivalence with the original seven-point instrument cannot be assumed, and direct comparisons of absolute SNAIL scores across studies should be interpreted cautiously. In addition, broader digital or informatics competencies were not assessed concurrently; therefore, the present study cannot determine whether the observed associations are specific to AI literacy or partly reflect competencies shared within the broader digital literacy context.

Fourth, although previous experience with AI-assisted tools was recorded, the study did not assess the frequency, intensity, purpose, type, or clinical context of actual AI use. In addition, workload was assessed in relation to routine clinical work during the preceding month, rather than workload experienced specifically during AI-assisted tasks. Furthermore, workload may vary across shifts and days, and the one-month recall period may have introduced recall bias and may not have fully captured short-term fluctuations in workload. Consequently, the observed associations should be interpreted as relationships between self-reported AI literacy and subjective workload, rather than as evidence that AI competency or actual AI use reduces workload associated with AI-assisted clinical work. Future studies should measure the frequency, purpose, and clinical context of actual AI-assisted tool use and assess workload during specific AI-assisted clinical tasks. Fifth, the three AI literacy dimensions were examined in separate adjusted models and were not mutually adjusted for; therefore, the observed associations should not be interpreted as the unique contribution of any single dimension. In addition, unmeasured factors such as patient acuity, staffing adequacy, direct-care intensity, and organizational characteristics may have contributed to residual confounding. Finally, the study was conducted at two regional hospitals within the same healthcare system in central Taiwan, which may limit the generalizability of the findings to other clinical settings and healthcare systems. As hospital site was not included in the regression models, potential clustering of workload perceptions within hospitals could not be evaluated. Differences in digital infrastructure, the extent of institutional AI implementation, nursing roles and responsibilities, and organizational culture across healthcare settings may influence both nurses’ AI literacy and their workload perceptions. Therefore, the observed associations may not be directly generalizable to institutions with different technological and organizational contexts.

## 5. Conclusions

AI literacy was selectively associated with nurses’ subjective workload, with the most consistent associations observed for lower temporal demand and frustration. More limited associations were found for physical demand and overall Raw TLX workload, while mental demand, effort, and performance did not show robust relationships. These findings suggest that AI literacy may be relevant to how nurses experience specific aspects of workload in increasingly technology-mediated clinical environments, rather than workload as a whole. These findings should be interpreted as associations between self-reported AI literacy and subjective workload, rather than evidence that AI competency or actual AI use reduces subjective workload. Future longitudinal and intervention studies incorporating measures of the frequency, purpose, and clinical context of actual AI-assisted tool use are needed to clarify these relationships.

## Figures and Tables

**Table 1:** Participant characteristics (N = 305).

Variable	Category	n	%
Gender	Male	37	12.1
	Female	268	87.9
Age group	18–29	93	30.5
	30–39	116	38.0
	40–49	65	21.3
	≥50	31	10.2
Education	Junior college	50	16.4
	Two-year technical	75	24.6
	Four-year technical	34	11.1
	University	115	37.7
	Master’s	31	10.2
Marital status	Unmarried/separated/widowed	152	49.8
	Married/cohabiting	153	50.2
Clinical ladder	N	62	20.3
	N1	51	16.7
	N2	117	38.4
	N3	43	14.1
	N4	32	10.5
Shift Type	Fixed day	166	54.4
	Fixed night	9	3.0
	Rotating three-shift	111	36.4
	Flexible/special	19	6.2
Supervisory status	Supervisor	48	15.7
	Non-supervisor	257	84.3
Administrative duties	No	105	34.4
	Yes	200	65.6
Primary clinical unit	Inpatient	117	38.4
	Special clinical	103	33.8
	Outpatient/primary	50	16.4
	LTC/community/other	35	11.5
Clinical nursing experience	1–5 years	73	23.9
	6–10 years	105	34.4
	11–15 years	39	12.8
	16–20 years	39	12.8
	>20 years	49	16.1
Previous AI-assisted tool use	Yes	159	52.1
	No	146	47.9

**Table 2:** Pearson correlations among the dimensions of AI literacy and subjective workload.

	Technical Understanding	Critical Appraisal	Practical Application
**AI literacy dimensions**			
Technical understanding	**-**		
Critical appraisal	0.810 (<0.001)	**-**	
Practical application	0.825 (<0.001)	0.881 (<0.001)	**-**
**Workload outcome**	**Technical understanding**	**Critical appraisal**	**Practical application**
Overall Raw TLX	−0.128 (0.025)	−0.075 (0.190)	−0.090 (0.119)
Mental demand	−0.056 (0.329)	0.007 (0.898)	−0.018 (0.749)
Physical demand	−0.147 (0.010)	−0.133 (0.020)	−0.161 (0.005)
Temporal demand	−0.193 (<0.001)	−0.148 (0.010)	−0.156 (0.006)
Performance	0.094 (0.103)	0.113 (0.048)	0.129 (0.024)
Effort	−0.052 (0.369)	0.020 (0.728)	0.000 (0.997)
Frustration	−0.146 (0.011)	−0.155 (0.007)	−0.156 (0.006)

Note: Values are Pearson correlation coefficients (r), with two-sided *p*-values in parentheses. N = 305. Higher scores in the NASA-TLX performance dimension indicate poorer perceived performance.

**Table 3:** Adjusted associations between AI literacy and subjective workload dimensions.

Workload Outcome	AI Literacy Dimension	ΔR^2^	B	95% CI for B	*p*	BH- Adjusted *p*
**Overall Raw TLX**	Technical understanding	0.021	−2.22	[−3.95, −0.50]	0.012	0.030
	Critical appraisal	0.009	−1.55	[−3.40, 0.30]	0.101	0.141
	Practical application	0.012	−1.91	[−3.82, 0.00]	0.050	0.088
**Mental demand**	Technical understanding	0.005	−1.63	[−4.31, 1.04]	0.230	0.285
	Critical appraisal	<0.001	−0.23	[−3.10, 2.63]	0.873	0.873
	Practical application	0.001	−0.77	[−3.73, 2.20]	0.611	0.642
**Physical demand**	Technical understanding	0.015	−3.04	[−5.69, −0.38]	0.025	0.052
	Critical appraisal	0.016	−3.36	[−6.19, −0.53]	0.020	0.047
	Practical application	0.023	−4.10	[−7.02, −1.18]	0.006	0.023
**Temporal demand**	Technical understanding	0.034	−4.90	[−7.81, −1.99]	0.001	0.020
	Critical appraisal	0.021	−4.04	[−7.17, −0.91]	0.012	0.030
	Practical application	0.024	−4.50	[−7.74, −1.26]	0.007	0.023
**Performance**	Technical understanding	0.006	3.04	[−1.30, 7.38]	0.169	0.221
	Critical appraisal	0.012	4.48	[−0.14, 9.10]	0.057	0.093
	Practical application	0.013	4.88	[0.10, 9.66]	0.045	0.087
**Effort**	Technical understanding	0.010	−1.94	[−4.15, 0.26]	0.084	0.126
	Critical appraisal	0.001	−0.61	[−2.98, 1.75]	0.610	0.642
	Practical application	0.002	−1.01	[−3.45, 1.44]	0.420	0.490
**Frustration**	Technical understanding	0.025	−4.87	[−8.27, −1.48]	0.005	0.023
	Critical appraisal	0.029	−5.51	[−9.14, −1.89]	0.003	0.021
	Practical application	0.031	−5.97	[−9.71, −2.23]	0.002	0.020

Note: Each AI literacy dimension was entered separately in a hierarchical regression model adjusted for gender, years of clinical nursing experience, primary clinical unit, shift type, supervisory status, and involvement in administrative duties. B = unstandardized regression coefficient; CI = confidence interval; ΔR^2^ = change in explained variance after adding the AI literacy dimension. BH-adjusted *p*-values were calculated using the Benjamini–Hochberg procedure across the 21 adjusted associations. Statistical significance and BH adjustment were based on unrounded *p*-values. Higher scores in the NASA-TLX performance dimension indicate poorer perceived performance.

## Data Availability

The data supporting the findings of this study are available from the corresponding author upon reasonable request, subject to ethical and institutional restrictions.

## Supplementary Materials

The following supporting information can be downloaded at: https://www.mdpi.com/article/10.3390/healthcare14193155/s1, Table S1: Sensitivity analyses of associations between AI literacy dimensions and subjective workload after additional adjustment for age, educational level, and previous AI tool experience.
