# Supplementary material for: Artificial Intelligence Literacy and Subjective Workload Among Clinical Nurses: A Cross-Sectional Study
Source: Healthcare (Basel). 2026 Sep 23;14(19):3155. doi: 10.3390/healthcare14193155 (PMC13664875; doi:10.3390/healthcare14193155)
Supplement: Supplementary file 1 [file healthcare-14-03155-s001.zip › healthcare-4522080-supplementary.pdf]

Table S1. Sensitivity analyses of associations between AI literacy dimensions and subjective workload after additional adjustment for age, educational level, and previous AI tool experience.

Workload outcome	AI literacy dimension	B	95% CI	p value	BH-FDR adjusted p
**Overall Raw TLX**	Technical understanding	−2.71	[−4.50 to −0.92]	0.003	0.013
	Critical appraisal	−1.83	[−3.80 to 0.14]	0.069	0.096
	Practical application	−2.37	[−4.44 to −0.30]	0.025	0.044
**Mental demand**	Technical understanding	−1.91	[−4.72 to 0.91]	0.183	0.214
	Critical appraisal	−0.13	[−3.20 to 2.94]	0.932	0.932
	Practical application	−0.81	[−4.05 to 2.43]	0.624	0.655
**Physical demand**	Technical understanding	−3.57	[−6.28 to −0.86]	0.01	0.023
	Critical appraisal	−3.49	[−6.44 to −0.54]	0.021	0.04
	Practical application	−4.41	[−7.52 to −1.30]	0.006	0.018
**Temporal demand**	Technical understanding	−5.34	[−8.36 to −2.32]	<.001	0.005
	Critical appraisal	−4.16	[−7.48 to −0.84]	0.014	0.03
	Practical application	−4.91	[−8.41 to −1.41]	0.006	0.018
**Performance**	Technical understanding	3.47	[−1.01 to 7.95]	0.129	0.159
	Critical appraisal	4.99	[0.13 to 9.85]	0.044	0.067
	Practical application	5.66	[0.53 to 10.79]	0.031	0.05
**Effort**	Technical understanding	−3.04	[−5.30 to −0.79]	0.008	0.022
	Critical appraisal	−1.48	[−3.96 to 0.99]	0.239	0.265
	Practical application	−2.17	[−4.78 to 0.44]	0.103	0.136
**Frustration**	Technical understanding	−5.87	[−9.40 to −2.34]	0.001	0.006
	Critical appraisal	−6.69	[−10.52 to −2.85]	<.001	0.005
	Practical application	−7.58	[−11.62 to −3.54]	<.001	0.005

Note: Each AI literacy dimension was examined in a separate regression model. Models were adjusted for sex, years of clinical nursing experience, primary clinical unit, shift type, supervisory status, involvement in administrative duties, age, educational level, and previous AI tool experience. The three AI literacy dimensions were not mutually adjusted. BH-FDR = Benjamini–Hochberg false discovery rate. Higher NASA-TLX performance scores indicate poorer perceived performance.
